# Decoding the Collagenome in Breast Cancer: Mechanotransduction, Microenvironment, and Translational Opportunities

**DOI:** 10.3390/ijms27156794

**Published:** 2026-07-29

**Authors:** Noelia Vigo-Díaz, Rubén López-Cortés, Laura Rodríguez-Silva, Marcelino Maneiro, Cristina Núñez

**Affiliations:** 1 Inorganic Chemistry Department, Institute for Research in Global Health and Sustainable Development (iTERRA), Faculty of Sciences, Campus Terra, University of Santiago de Compostela, 27002 Lugo, Spain; noelia.vigo@usc.es (N.V.-D.); laura.rodriguez@usc.es (L.R.-S.); marcelino.maneiro@usc.es (M.M.); 2 Research Unit, Hospital Universitario Lucus Augusti (HULA), Servizo Galego de Saúde (SERGAS), 27002 Lugo, Spain; ruben.lopez.cortes@sergas.es

**Keywords:** breast cancer (BC), extracellular matrix (ECM), collagen remodelling, tumour microenvironment (TME), mechanotransduction

## Abstract

Breast cancer (BC) progression is strongly influenced by the extracellular matrix (ECM), whose remodelling regulates tumour growth, invasion, metastasis, immune modulation, and therapeutic response. Among ECM components, collagens have emerged as both structural proteins and active mediators of mechanotransduction, stromal interactions, and tumour cell behaviour. This narrative review analyses collagen families and collagen-associated proteins implicated in BC, integrating evidence on their expression patterns, biological functions, clinical significance, and translational potential. We examine fibrillar and non-fibrillar collagens, including fibril-associated collagens with interrupted triple helices (FACITs), membrane-associated collagens with interrupted triple helices (MACITs), basement membrane (BM) collagens, and multiplexins, together with their interactions with cancer-associated fibroblasts (CAFs), immune cells, and signalling pathways involved in tumour progression. Alterations in collagen composition, organization, crosslinking, and degradation regulate ECM stiffness, epithelial–mesenchymal transition (EMT), invasion, metastatic dissemination, and therapy resistance. Several collagen types and collagen-derived fragments also show promise as prognostic biomarkers and therapeutic targets, particularly in aggressive BC subtypes such as human epidermal growth factor receptor 2 (HER2)-positive and triple-negative breast cancer (TNBC). Overall, this review highlights the collagenome as a dynamic component of the breast tumour microenvironment (TME) and supports collagen-informed strategies for improved patient stratification and targeted therapies.

## 1. Introduction

Solid tumours are complex multicellular entities characterized by the coexistence of malignant cells and a heterogeneous microenvironment. Beyond cancer cells, the TME comprises immune and inflammatory cells, fibroblasts (including CAFs), endothelial and lymphatic cells, adipocytes, and ECM. The ECM is not a passive scaffold. Instead, it constitutes a dynamic and highly organized molecular network that continuously remodels its composition, architecture, and mechanical properties, thereby influencing tumour evolution. Aberrant ECM remodelling is now recognized as a hallmark of many solid cancers, and in BC, it strongly influences tumour initiation, local invasion, metastatic dissemination, therapeutic response, and relapse [1,2].

Throughout this review, the term collagenome refers to the complete repertoire of collagen molecules that constitute the breast tumour ECM, including their supramolecular organization, biologically active collagen-derived fragments generated during ECM remodelling, and their functional interactions with collagen-binding receptors and associated signalling pathways. Thus, the collagenome is considered not merely as the collection of collagen genes or proteins, but as a dynamic structural and signalling network that actively regulates tumour progression, stromal communication, immune modulation, and therapeutic response. Although proteins containing collagen-like domains are mentioned where relevant, the primary focus of this review is on human collagen families and their biological roles in breast cancer.

Collagen-rich matrices exhibit marked structural heterogeneity, and increasing evidence indicates that collagen architecture actively regulates tumour invasion through mechanotransduction. For example, a Yes-associated protein (YAP)-centred mechanotransduction circuit couples collagen organization with collective BC invasion, reinforcing the concept that collagen architecture functions as an instructive regulator of tumour progression rather than merely providing structural support [2,3]. Likewise, physiological tissue-remodelling processes, such as postpartum mammary gland involution, further support the concept that ECM remodelling contributes to tumour progression by creating a transient pro-remodelling microenvironment [4].

**Figure 1:**
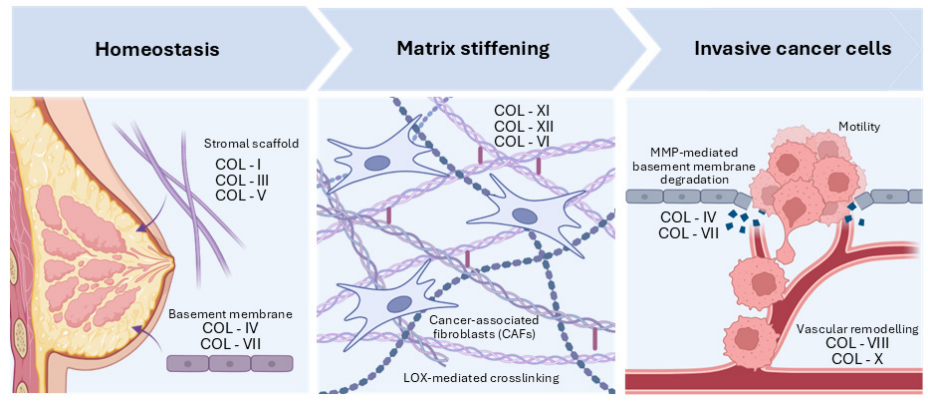
Schematic overview of collagen-mediated ECM remodelling during BC progression. Normal mammary tissue displays organized interstitial and BM collagens that maintain ECM homeostasis. During tumour progression, CAFs, progressive collagen deposition, collagen fibre remodelling, LOX-mediated crosslinking, and BM remodelling promote ECM stiffening, aberrant mechanotransduction, tumour invasion, and metastatic dissemination. The collagen types indicated in each panel are representative examples selected on the basis of their predominant experimentally and/or clinically supported functional involvement in the biological processes illustrated, rather than their exclusive expression, localization, or stage-specific occurrence. Their inclusion does not imply exclusive expression, localization, degradation, or stage-specific occurrence, since several collagen types participate in multiple and overlapping aspects of BC progression. The schematic integrates available evidence on collagen deposition and stromal expansion, collagen fibre organization, ECM stiffening, BM remodelling, and vascular invasion, as described in previous studies [2,3,5,6,7]. Created in BioRender. Vigo, N. (2026) https://BioRender.com/vmoojta (accessed on 3 March 2026). Abbreviations: LOX, lysyl oxidase; MMP, matrix metalloproteinase; COL, collagen (Roman numerals indicate the corresponding collagen type).

Collagen remodelling is coordinated through synthesis, enzymatic processing, crosslinking, and proteolytic degradation. Matrix metalloproteinases (MMPs) regulate collagen turnover and the release of bioactive ECM fragments, whereas lysyl oxidase (LOX) family enzymes promote collagen crosslinking and tissue stiffening, thereby contributing to mechanotransduction, tumour progression, and therapy resistance. In parallel, CAFs represent the major collagen-producing cells within BC, and play a central role in ECM remodelling, stromal organization, and tumour–stroma communication [5,6,7,8]. Together, these interconnected processes progressively transform the breast ECM from a homeostatic scaffold into a mechanically active and invasion-promoting microenvironment, as summarized in Figure 1.

**Figure 2:**
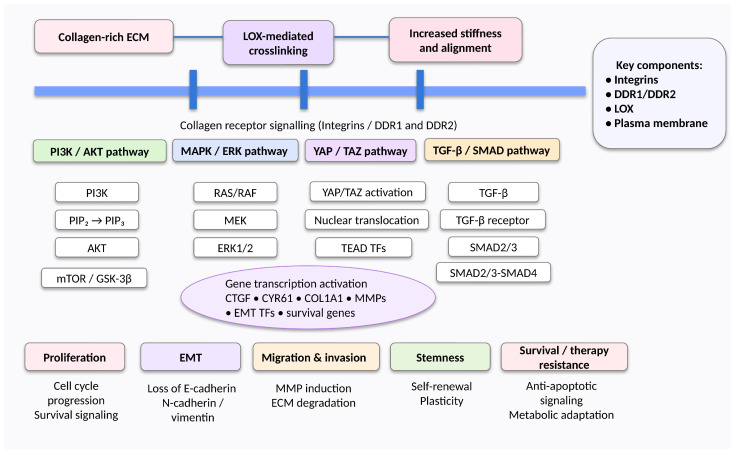
Mechanotransduction pathways induced by collagen remodelling in breast cancer. Collagen deposition by CAFs, together with LOX-mediated collagen crosslinking, promotes ECM stiffening and collagen fibre alignment. These biochemical and mechanical cues are sensed through collagen-binding receptors, including integrins and discoidin domain receptors (DDR1 and DDR2), leading to the activation of interconnected mechanotransduction pathways, including PI3K/AKT, MAPK/ERK, YAP/TAZ, and TGF-β/SMAD signalling. The coordinated activation of these pathways promotes transcriptional programmes associated with tumour progression, including increased proliferation, EMT, migration, invasion, stemness, metabolic adaptation, and therapy resistance. Abbreviations: ECM, extracellular matrix; CAFs, cancer-associated fibroblasts; LOX, lysyl oxidase; DDR, discoidin domain receptor; PI3K, phosphoinositide 3-kinase; AKT, protein kinase B; mTOR, mechanistic target of rapamycin; GSK-3β, glycogen synthase kinase-3β; MAPK, mitogen-activated protein kinase; ERK, extracellular signal-regulated kinase; YAP, Yes-associated protein; TAZ, transcriptional co-activator with PDZ-binding motif; TEAD, TEA domain transcription factor; TGF-β, transforming growth factor beta; SMAD, mothers against decapentaplegic homolog; CTGF, connective tissue growth factor; CYR61, cysteine-rich angiogenic inducer 61 (CCN1); COL1A1, collagen type I alpha 1 chain; MMPs, matrix metalloproteinases; EMT, epithelial–mesenchymal transition.

Beyond its structural role, collagen remodelling influences multiple biological processes involved in BC progression. Alterations in collagen organization regulate tissue mechanics, mechanotransduction, metabolic adaptation, stromal communication, and clinically relevant phenotypes associated with prognosis and therapeutic response. The principal signalling pathways activated by collagen remodelling are summarized in Figure 2 [2,3,7].

Collagen alterations are also increasingly recognized as clinically relevant biomarkers. Both tissue-based collagen signatures and circulating markers reflecting ECM turnover have shown potential for prognostic stratification and monitoring stromal activation, supporting the concept that collagen remodelling may be exploited through imaging, tissue analysis, and liquid biopsy approaches [8,9,10].

Altogether, these observations support the view that the collagenome constitutes a dynamic structural and signalling network integrating collagen diversity, ECM remodelling, and collagen-mediated cellular communication. Because collagen remodelling may differ across clinicopathological BC subgroups defined by hormone receptor and HER2 status, available evidence should be interpreted according to the strength and type of supporting studies. Table 1 summarizes the principal collagen-associated features reported for the main BC subtypes.

Before discussing the individual collagen families, it is important to emphasize that the evidence available for each collagen type derives from different experimental approaches. Throughout this review, we distinguish, whenever possible, whether the reported findings originate from transcriptomic analyses, proteomic studies, immunohistochemistry, ECM imaging, serum-based assays, or functional experimental models. This distinction is particularly relevant because changes in collagen gene expression do not necessarily reflect collagen protein abundance, ECM organization, or biological function.

Accordingly, this narrative review examines the spectrum of collagen types implicated in BC, integrating recent evidence regarding their cellular sources, biological functions, mechanistic contributions, and translational potential [2,5,9,10,18]. Given the remarkable diversity of collagen molecules within the breast TME, a collagen-type-resolved framework is required to understand their distinct biological functions.

Although collagens are traditionally classified according to their supramolecular organization, increasing evidence indicates that their functions extend far beyond structural support. Different collagen subtypes contribute differentially to ECM stiffness, mechanotransduction, immune regulation, angiogenesis, metastatic dissemination, and therapeutic resistance in a context-dependent manner. Human collagens are currently classified into several supramolecular subgroups, including fibrillar collagens, FACITs, BM collagens, network-forming collagens, MACITs, and multiplexins [22,23]. Their principal characteristics and reported implication in BC are summarized in Table 2.

**Figure 3:**
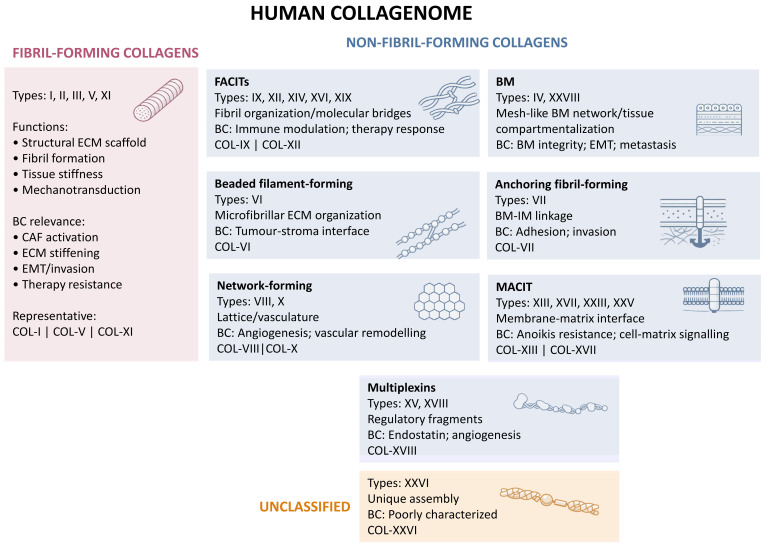
Classification of the human collagenome and its functional relevance in breast cancer. Human collagens are classified into fibril-forming, non-fibrillar, and unclassified collagen families according to their structural organization and biological functions. Fibril-forming collagens constitute the major structural framework of the ECM, whereas non-fibrillar collagens comprise specialised subfamilies involved in basement membrane organization, fibril regulation, cell–matrix interactions, and tissue homeostasis. The lower panel summarizes the principal biological functions of each collagen family together with representative collagen types and their reported relevance in BC, including extracellular matrix remodelling, tumour progression, EMT, angiogenesis, invasion, immune modulation, and therapy resistance. Abbreviations: BC, breast cancer; ECM, extracellular matrix; BM, basement membrane; IM, interstitial matrix; CAF, cancer-associated fibroblast; EMT, epithelial–mesenchymal transition; FACIT, fibril-associated collagens with interrupted triple helices; MACIT, membrane-associated collagens with interrupted triple helices; COL, collagen (Roman numerals indicate the corresponding collagen type).

Figure 3 provides a schematic overview of this hierarchical classification and illustrates the principal functional implications of each collagen subgroup in BC. This framework serves as the basis for the collagen-type-resolved discussion developed throughout the following sections [22,23,30].

## 2. The Collagen Mechanobiology Axis in BC

### 2.1. Collagen Accumulation and ECM Expansion

The earliest stages of ECM remodelling in BC are characterized by progressive collagen accumulation and expansion of the stromal compartment. Instead of representing a passive consequence of tumour growth, excessive collagen deposition actively reshapes the TME, creating a mechanically dynamic niche that promotes malignant progression. This process is driven primarily by CAFs, which become persistently activated through reciprocal interactions with tumour cells, inflammatory cytokines, and transforming growth factor-β (TGF-β). Activated CAFs have been identified by transcriptomic, proteomic, and immunohistochemical studies as the principal producers of fibrillar collagens, particularly collagen I, III, and V. These studies also demonstrate increased transcript and protein expression of matrix-remodelling enzymes associated with CAF activation, establishing a self-reinforcing fibrotic programme that progressively modifies ECM composition and architecture [50].

Collagen accumulation is accompanied by profound changes in the cellular composition of the tumour stroma. In addition to CAFs, tumour-associated adipocytes, endothelial cells, infiltrating immune cells, and, in some cases, BC cells themselves contribute to matrix remodelling by producing collagen molecules or by secreting cytokines and growth factors that further stimulate stromal activation. Consequently, collagen deposition should be regarded as the integrated output of multiple cellular populations rather than the exclusive product of fibroblasts. This reciprocal communication establishes a positive feedback loop in which tumour cells stimulate stromal collagen production, while the altered ECM provides biochemical and biomechanical signals that further enhance tumour aggressiveness [109].

Histopathological analyses, together with second harmonic generation (SHG) imaging studies, have consistently shown that the transition from normal mammary tissue to invasive carcinoma is associated with progressive stromal fibrosis, increased collagen deposition, higher collagen fibre density, and expansion of desmoplastic regions. These structural changes profoundly modify the physical properties of the breast TME. They are also closely related to mammographic breast density, one of the strongest independent risk factors for BC development and progression. Accordingly, mammographic breast density should be interpreted as a macroscopic surrogate of collagen-rich stromal remodelling rather than a direct indicator of tumour aggressiveness. Importantly, collagen accumulation alone does not fully explain the biological consequences of ECM remodelling. Instead, the quantity of collagen provides the structural substrate upon which subsequent architectural reorganization, enzymatic crosslinking, and mechanical stiffening take place. Thus, collagen abundance represents the first hierarchical step in a cascade of biomechanical events that ultimately governs tumour behaviour [110].

Experimental studies have demonstrated that excessive collagen deposition increases the probability of cell–matrix interactions by enlarging the available collagen surface that can be recognized by collagen receptors, including integrins and DDRs. These interactions not only reinforce cell adhesion, but also facilitate the transmission of mechanical forces from the extracellular environment to the cytoskeleton, thereby priming tumour cells for mechanotransduction. Nevertheless, collagen abundance alone is insufficient to generate the highly invasive phenotype observed in advanced BC. The biological impact of collagen deposition critically depends on the spatial organization of collagen fibres and on the degree of enzymatic crosslinking that determines matrix stiffness. Therefore, collagen accumulation should be viewed as the initiating event of a sequential mechanobiological process rather than as an isolated determinant of tumour progression [111].

### 2.2. Collagen Fibre Alignment and Architectural Remodelling

SHG imaging, multiphoton microscopy, and computational image-analysis studies have demonstrated that the three-dimensional organization of collagen fibres, rather than collagen abundance alone, is a major determinant of BC progression. During tumour evolution, these imaging approaches reveal that collagen fibres undergo profound architectural reorganization, becoming progressively straighter, thicker, and more anisotropically aligned. These structural alterations transform the ECM from a compliant scaffold into a highly organized mechanical network that actively regulates tumour cell behaviour. Therefore, collagen architecture has emerged as an important prognostic feature, complementing conventional molecular biomarkers and histopathological parameters [112].

One of the best-characterized examples of matrix imaging in BC is the progressive appearance of Tumour-Associated Collagen Signatures (TACSs). These collagen architectural patterns were originally identified by SHG microscopy and subsequently validated using complementary multiphoton imaging approaches. TACS-1 is characterized by locally increased collagen deposition surrounding early tumour lesions, whereas TACS-2 consists of stretched collagen fibres arranged tangentially around the expanding tumour mass. As invasion progresses, collagen fibres become radially aligned perpendicular to the tumour boundary, giving rise to the TACS-3 phenotype, which is consistently associated with enhanced tumour invasion, lymph node metastasis, and poor clinical outcome. More recently, additional collagen architectural patterns (TACS-4 to TACS-8) have been described, reflecting the increasing spatial complexity of the invasive front, and further emphasizing that collagen organization evolves continuously during BC progression [112].

The biological significance of collagen alignment resides in its ability to provide contact-guidance cues for migrating tumour cells. Unlike randomly organized collagen networks, aligned collagen bundles generate preferential migration tracks that facilitate persistent and directional cell movement toward blood vessels and surrounding tissues. This phenomenon is driven by the physical orientation of collagen fibrils rather than by biochemical gradients alone, allowing tumour cells to migrate collectively or individually along pre-existing collagen highways. Accordingly, collagen alignment not only promotes local invasion, but also facilitates intravasation and the early stages of metastatic dissemination. This concept is supported by experimental mechanobiology models combining collagen-matrix engineering and live-cell imaging, which have consistently demonstrated that increased collagen anisotropy enhances cell polarity, directional migration, and invasive capacity, highlighting ECM architecture as an active regulator of tumour dissemination rather than a passive structural framework [50].

Collagen fibre orientation also profoundly influences the tumour immune microenvironment. Recent imaging studies together with functional experimental models have demonstrated that highly aligned collagen bundles may function as a physical barrier restricting immune-cell infiltration into tumour nests. In particular, collagen organization mediated by discoidin domain receptor 1 (DDR1) promotes the formation of densely packed collagen architectures that limit T-cell access to malignant epithelial regions. Experimental blockade of DDR1 disrupts collagen alignment, increases immune-cell penetration, and enhances antitumour immune responses, indicating that collagen architecture contributes not only to tumour invasion, but also to immune escape. These observations reinforce the concept that ECM organization represents a dynamic regulator of both tumour progression and antitumour immunity [112].

The clinical relevance of collagen architecture has increased considerably owing to advances in imaging technologies. SHG microscopy, polarized light microscopy, multiphoton microscopy, and computational image analysis now enable quantitative assessment of collagen alignment, fibre orientation, anisotropy, and microstructural organization in tumour specimens. These approaches have shown that collagen architectural parameters frequently outperform collagen quantity alone in predicting disease-free survival, metastatic risk, and therapeutic response. Accordingly, collagen alignment is increasingly recognized as a promising imaging biomarker that captures biomechanical properties of the TME not reflected by conventional histopathology [112]. Notably, these imaging approaches characterize collagen architecture and fibre organization rather than collagen gene expression or protein abundance, providing complementary information on ECM biomechanics.

Importantly, collagen alignment should not be considered an isolated phenomenon, but rather the intermediate step linking collagen deposition with matrix stiffening and mechanotransduction. As collagen fibres become progressively aligned, they provide an optimal substrate for enzymatic crosslinking by LOX family members, resulting in increased ECM rigidity and enhanced transmission of mechanical forces to tumour cells. Therefore, architectural remodelling of collagen constitutes the structural bridge between ECM expansion and activation of intracellular mechanosensitive signalling pathways that drive BC progression [50].

### 2.3. LOX-Mediated Collagen Crosslinking and ECM Stiffening

Although collagen deposition and fibre alignment establish the structural framework of the TME, the acquisition of its biomechanical properties is primarily determined by collagen crosslinking. This process is catalysed by the LOX family of copper-dependent amine oxidases (LOX and LOXL1–LOXL4), which generate covalent intermolecular crosslinks between collagen molecules, thereby increasing fibrillar stability, tensile strength, and resistance to proteolytic degradation. Thus, collagen crosslinking transforms the ECM into a mechanically rigid scaffold that profoundly influences tumour cell behaviour. Growing evidence indicates that this increase in matrix stiffness is not merely a consequence of tumour progression, but an active driver of BC initiation, invasion, and metastatic dissemination [50].

Transcriptomic and mechanistic experimental studies have shown that LOX expression is tightly regulated by several tumour-associated stimuli, particularly hypoxia and transforming growth factor-β (TGF-β). Under hypoxic conditions, stabilization of hypoxia-inducible factor-1α (HIF-1α) promotes LOX transcription, whereas activated CAFs further increase LOX production through paracrine signalling. These mechanisms generate a positive feedback loop in which fibrosis, collagen deposition, and matrix stiffening continuously reinforce one another during tumour progression. Biochemical and ECM imaging studies indicate that increased collagen crosslinking protects collagen fibres from MMP-mediated degradation, thereby contributing to the persistence of a dense desmoplastic microenvironment [110].

Biomechanical studies using engineered collagen matrices have demonstrated that the mechanical consequences of collagen crosslinking extend beyond increased tissue rigidity. Crosslinked collagen fibres exhibit higher resistance to deformation and transmit mechanical forces more efficiently to adherent cells. This enhanced force transmission promotes focal adhesion maturation, actomyosin contractility, and cytoskeletal tension, enabling tumour cells to continuously sense changes in ECM mechanics. Thus, collagen crosslinking represents the crucial physical event that converts structural ECM remodelling into biologically relevant mechanical signals. In BC, experimental studies have demonstrated that increased matrix stiffness enhances cell proliferation, survival, EMT, migration, and invasion while simultaneously promoting angiogenesis, stemness, and resistance to systemic therapies [110].

Mechanobiology studies have demonstrated that ECM stiffening initiates mechanotransduction through integrin-dependent signalling pathways. Increased collagen rigidity promotes integrin clustering and focal adhesion assembly, resulting in the activation of FAK, Src family kinases, and RhoA/ROCK-mediated actomyosin contractility. These signalling cascades ultimately regulate the nuclear localization of the mechanosensitive transcriptional co-activators YAP and transcriptional co-activator with PDZ-binding motif (TAZ), which coordinate gene expression programmes involved in proliferation, ECM remodelling, stem-cell maintenance, and therapeutic resistance. Importantly, YAP activation also induces the transcription of ECM-remodelling genes, including collagen-modifying enzymes, thereby establishing a feed-forward loop that further enhances matrix stiffening and tumour progression [3].

Functional organoid-based mechanobiology studies have recently shown that collagen I does not simply provide a permissive substrate for invasion, but actively participates in a reciprocal mechanical dialogue with tumour cells. Contact with collagen I induces YAP activation in basal-like BC cells, whereas activated YAP promotes transcriptional programmes that increase collagen remodelling and fibre alignment, generating localized regions of elevated mechanical tension that further amplify YAP signalling. This self-reinforcing mechanotransduction circuit facilitates leader-cell formation and collective invasion, providing direct experimental evidence that collagen remodelling and intracellular mechanosignalling constitute an integrated biological process rather than independent events [3].

The translational implications of these findings are considerable. Preclinical experimental studies suggest that pharmacological inhibition of LOX activity, interference with collagen crosslinking, or blockade of stiffness-sensitive signalling pathways may reduce tumour fibrosis, improve drug penetration, and enhance the efficacy of chemotherapy, targeted therapies, and immunotherapy. Although most of these strategies remain in preclinical or early clinical development, they support the concept that targeting tumour biomechanics represents a promising therapeutic approach complementary to conventional anticancer treatments [109].

### 2.4. Collagen Sensing Through Integrins and Discoidin Domain Receptors

Functional mechanobiology studies have demonstrated that the biological effects of collagen remodelling ultimately depend on the ability of cells to sense changes in ECM composition and mechanics. This function is mediated primarily by two major classes of collagen receptors: integrins and DDRs. Although both receptor families recognize collagen molecules, they bind distinct collagen motifs and activate complementary signalling pathways, enabling cells to integrate biochemical and biomechanical information from the surrounding ECM. Rather than functioning independently, increasing evidence indicates that integrins and DDRs cooperate to orchestrate cellular responses to collagen remodelling, thereby regulating tumour progression, immune evasion, and therapeutic resistance [111].

Functional cell biology and mechanobiology studies have established that integrins constitute the principal mechanoreceptors responsible for sensing matrix stiffness. In BC, collagen-binding integrins, particularly α1β1, α2β1, α10β1, and α11β1, interact with fibrillar collagens and promote the assembly of focal adhesions, which mechanically couple the ECM to the actin cytoskeleton. Increased collagen density, fibre alignment, and matrix stiffening enhance integrin clustering, thereby promoting the recruitment of FAK, Src family kinases, and integrin-linked kinase (ILK). These signalling complexes activate downstream pathways, including PI3K/AKT, MAPK/ERK, and RhoA/ROCK, leading to cytoskeletal remodelling, increased cellular contractility, and enhanced proliferation, migration, and survival. As a result, integrins function as the primary sensors that convert ECM stiffness into intracellular biochemical signals, initiating mechanotransduction programmes that promote tumour progression [113].

Unlike integrins, DDRs are receptor tyrosine kinases that are activated directly by native triple-helical collagen molecules. Transcriptomic analyses together with immunohistochemical studies indicate that DDR1 is predominantly expressed in epithelial tumour cells, whereas DDR2 is mainly associated with stromal fibroblasts and mesenchymal-like tumour cells. Following collagen binding, DDRs undergo a slow but sustained activation that differs from the rapid signalling characteristic of integrins. This prolonged signalling regulates multiple biological processes, including ECM remodelling, epithelial differentiation, cell survival, and migration. Recent evidence further suggests that DDR1 also functions as a mechanosensor capable of responding to extracellular stiffness and cytoskeletal tension, thereby extending its role beyond collagen recognition to the regulation of tissue mechanics [114].

DDR1 and DDR2 exhibit distinct but complementary functions during BC progression. DDR1 contributes to tumour growth by promoting cell survival, maintaining epithelial plasticity, and regulating collagen organization. Remarkably, matrix imaging studies combined with functional experimental models have recently demonstrated that DDR1-mediated alignment of collagen fibres generates a dense peritumoural collagen barrier that limits T-cell infiltration, thereby facilitating immune exclusion and reducing antitumour immunity. These imaging and functional studies also provide a mechanistic explanation for the current evidence that collagen architecture and DDR1-mediated matrix organization may influence responsiveness to immune checkpoint blockade by restricting cytotoxic T-cell access to tumour cells. Conversely, DDR2 plays a central role in tumour invasion by stabilizing the EMT transcription factor SNAIL1 through Src-dependent ERK2 signalling, promoting EMT, and facilitating migration through collagen-rich stroma. These observations highlight that collagen receptors not only regulate tumour cell behaviour, but also shape the immune and stromal compartments of the TME [50].

Current evidence from functional experimental models indicates that integrins and DDRs should not be viewed as independent signalling systems, but rather as components of an integrated collagen-sensing network. Integrin activation rapidly transduces mechanical forces generated by ECM stiffening, whereas DDR signalling provides sustained biochemical responses to collagen engagement. Crosstalk between these receptors amplifies mechanotransduction, coordinates cytoskeletal remodelling, and regulates transcriptional programmes associated with proliferation, invasion, stemness, and therapeutic resistance. This coordinated signalling enables tumour cells to continuously adapt to dynamic changes in collagen abundance, fibre organization, and matrix stiffness throughout tumour progression [111].

Activation of collagen receptors ultimately converges on several evolutionarily conserved signalling pathways, including FAK/Src, PI3K/AKT, MAPK/ERK, and the Hippo effectors YAP/TAZ. These downstream pathways integrate extracellular mechanical information with transcriptional responses controlling cell proliferation, metabolic adaptation, ECM remodelling, and metastatic dissemination. More importantly, recent organoid-based mechanobiology studies and in vivo experimental models have demonstrated that YAP activation further stimulates collagen remodelling and LOX expression, thereby reinforcing matrix stiffening and generating a self-sustaining feed-forward circuit that promotes collective invasion. For this reason, collagen receptors represent the critical molecular interface linking ECM remodelling with intracellular mechanotransduction, and constitute attractive therapeutic targets for disrupting tumour–stroma interactions [3].

### 2.5. Downstream Mechanotransduction Pathways: Integrating Biochemical and Biomechanical Signalling

Functional mechanobiology studies have demonstrated that mechanical information generated by collagen remodelling is ultimately translated into coordinated intracellular signalling networks that regulate virtually every aspect of tumour biology. Rather than functioning as isolated cascades, mechanotransduction pathways constitute a highly interconnected signalling system in which integrins, DDRs, growth factor receptors, and cytoskeletal mechanosensors cooperate to integrate biochemical and biomechanical cues derived from the ECM. This signalling network enables tumour cells to continuously adapt to changes in collagen abundance, fibre architecture, and matrix stiffness, thereby promoting phenotypic plasticity during BC progression [115].

Functional cell biology studies have established that one of the earliest intracellular events following collagen receptor activation is the assembly of focal adhesions. Engagement of collagen-binding integrins induces rapid recruitment and autophosphorylation of FAK, which serves as the principal signalling hub connecting extracellular mechanical stimuli with intracellular kinase cascades. Biochemical and functional experimental studies have shown that activated FAK associates with Src family kinases to regulate focal adhesion turnover, cytoskeletal remodelling, and actomyosin contractility, while simultaneously initiating downstream PI3K/AKT and MAPK/ERK signalling. Together, these pathways promote cell-cycle progression, survival, metabolic adaptation, and migration, thereby enabling tumour cells to respond efficiently to mechanical changes within the surrounding stroma. Persistent activation of FAK/Src signalling has been consistently associated with increased invasiveness, metastatic dissemination, and poor clinical outcome in BC, underscoring its central role in collagen-dependent mechanotransduction [113].

Mechanical signalling further converges on the Rho family of small GTPases, particularly RhoA, which regulates actomyosin contractility through activation of Rho-associated coiled-coil kinase (ROCK). Increased collagen stiffness enhances RhoA/ROCK activity, leading to stress-fibre formation, elevated intracellular tension, and maturation of focal adhesions. These cytoskeletal rearrangements reinforce integrin signalling and establish a positive mechanical feedback loop in which increasing matrix stiffness promotes greater cellular contractility, which in turn further remodels the through enhanced traction forces exerted by both tumour cells and CAFs. Therefore, ECM remodelling and intracellular force generation become tightly coupled processes that continuously reinforce one another throughout tumour progression [110].

Among the downstream effectors of mechanotransduction, the Hippo pathway transcriptional co-activators YAP and TAZ have emerged as master regulators of mechanical signalling. Under compliant matrix conditions, YAP and TAZ remain predominantly cytoplasmic and transcriptionally inactive. In contrast, increasing ECM stiffness, collagen crosslinking, and actomyosin tension promote their nuclear translocation, where they interact with TEAD transcription factors to regulate genes involved in proliferation, ECM remodelling, epithelial plasticity, stem-cell maintenance, and therapeutic resistance. Unlike transient kinase activation, YAP/TAZ signalling provides sustained transcriptional adaptation to persistent mechanical stimulation, allowing tumour cells to acquire long-term phenotypic changes in response to alterations in the ECM [3].

Recent experimental studies have demonstrated that YAP signalling is not only activated by collagen remodelling, but also actively contributes to its maintenance. Nuclear YAP promotes the expression of ECM proteins, LOX family members, and multiple CAF-derived profibrotic mediators, thereby reinforcing collagen deposition, fibre alignment, and matrix stiffening. This feed-forward circuit establishes a self-perpetuating biomechanical programme in which ECM remodelling continuously enhances intracellular mechanotransduction, while activated mechanotransduction pathways further remodel the extracellular environment. Such reciprocal regulation has emerged as one of the defining hallmarks of tumour mechanobiology, and provides a mechanistic explanation for the progressive increase in tissue stiffness observed during BC evolution [3].

Importantly, mechanotransduction does not operate independently of canonical oncogenic signalling pathways. Mechanical activation of integrins and DDRs cooperates with receptor tyrosine kinases such as epidermal growth factor receptor (EGFR), HER2, and TGF-β receptors, amplifying proliferative and survival signals through shared downstream effectors. As a results, collagen remodelling not only influences tumour mechanics, but also modifies the intensity and duration of growth factor signalling, thereby contributing to tumour heterogeneity and variable therapeutic responses. This extensive crosstalk explains why alterations in ECM composition frequently affect sensitivity to chemotherapy, endocrine therapy, HER2-targeted agents, and immune checkpoint inhibitors, even in the absence of additional genetic alterations [115].

Overall, these observations support the concept that collagen-dependent mechanotransduction represents an integrated signalling network rather than a collection of independent pathways. Mechanical cues generated by collagen accumulation, fibre alignment, and matrix stiffening are sensed through collagen receptors and transmitted via interconnected FAK/Src, PI3K/AKT, MAPK/ERK, RhoA/ROCK, and YAP/TAZ signalling pathways, ultimately regulating tumour cell plasticity, stromal interactions, and disease progression. However, these mechanobiological responses are not mediated uniformly by all collagens. Instead, they depend on the specific structural, biochemical, and signalling properties of individual collagen molecules. Accordingly, the following sections examine the principal collagen families and discuss their distinct contributions to ECM remodelling and BC biology.

## 3. Fibrillar Collagens

Fibrillar collagens are the principal structural determinants of ECM architecture and mechanical properties in BC. Alterations in their abundance, organization, and crosslinking directly influence tissue stiffness, tumour–stroma interactions, and invasive behaviour.

In healthy breast tissue, fibrillar collagens are tightly organized within interstitial matrix (IM) and BM compartments. However, tumour progression is accompanied by profound alterations in collagen architecture and spatial organization.

The collagen composition of the ECM is highly tissue-specific and spatially heterogeneous. In healthy breast tissue, the expression of collagens I, III, and V depends on fibroblasts located in IM, whereas collagens IV and VII are mainly produced by epithelial cells associated with the BM. Multiple stromal and tumour-associated cell populations can contribute to collagen production. Thus, collagen production is not restricted to resident breast cells, as multiple tumour-associated and stromal cell populations also contribute to ECM remodelling [30].

Of note, breast density is a clinically relevant parameter associated with BC risk and progression. In addition, the ECM actively regulates stromal-cell and immune-cell behaviour [116].

### 3.1. Collagen I

In healthy breast tissue, collagen I fibrils display a highly organized parallel arrangement. During BC progression, CAFs promote excessive collagen I deposition and collagen crosslinking, leading to progressive ECM disorganization and increased tissue stiffness. These alterations contribute to tumour progression and immune evasion, and collagen I remodelling is currently being explored as an imaging- and histology-based biomarker for BC stratification and prognostic assessment [24,117].

As a major determinant of ECM stiffness and mechanotransduction, collagen I actively shapes tumour cell adhesion, protease secretion, and invasive behaviour. Increased collagen I deposition enhances MMP expression, promotes cell–matrix interactions, and facilitates metastatic dissemination [25]. However, another study reported that collagen I induced apoptosis in luminal BC cells, but not in the basal-like subtype, likely due to differential MMP-14 expression [26]. These apparently conflicting observations likely reflect the context-dependent and subtype-specific functions of collagen I during BC progression.

Functional experimental studies suggest that *COL1A1* may act as an oncogenic driver in BC, as experimental downregulation of *COL1A1* inhibits tumour-associated fibroblast activation and ECM remodelling [27]. Together, these findings reinforce the concept that ECM remodelling actively shapes tumour cell behaviour and adaptive tumour progression. Recent studies have shown that low *COL1A1* mRNA and protein levels correlate with unfavourable tumour characteristics in BC and may predict chemotherapy sensitivity [12]. These findings suggest that collagen I does not act as a universally pro-tumoural cue, but rather exerts subtype-dependent effects shaped by receptor expression, protease activity, and stromal context.

### 3.2. Collagen II

The functional interplay between the ECM and tumour cell behaviour is further illustrated by the example of another fibrillar collagen, collagen II. Transcriptomic analyses have shown that increased *COL2A1* mRNA expression is associated with reduced response to neoadjuvant anti-HER2 therapy in HER2-positive BC, even when combined with phosphoinositide 3-kinase (PI3K) inhibitors. Mechanistically, this resistance appears to involve activation of the integrin-β1/proto-oncogene tyrosine-protein kinase Src (Src) signalling pathway, which promotes tumour cell motility and proliferation [14]. Notably, resistance was reversed following treatment with a collagen synthesis inhibitor, further supporting the direct contribution of ECM remodelling to therapeutic resistance.

### 3.3. Collagen III

Transcriptomic analyses indicate that *COL1A1* and *COL3A1* mRNA expression levels are low in normal tissue and benign lesions, but increase during tumour progression, likely reflecting enhanced ECM turnover [28]. However, accumulating evidence indicates that collagen III exerts context-dependent functions. Dormant tumours from lymph node-negative head and neck carcinoma patients displayed collagen III-enriched matrices [32], consistent with BC murine studies showing increased tumour formation in *COL3A1*-deficient mice [33]. Mechanistically, collagen III was shown to suppress key metastatic processes in experimental models.

Nevertheless, other studies suggest that collagen III may exert pro-migratory effects under specific microenvironmental conditions. For example, another study found that the presence of collagen α1(III) chain in conditioned medium from bone mesenchymal stem-cells promoted BC-cell migration in Transwell assays [29]. Notably, this response was maximal at two hours and then declined rapidly.

In contrast, the presence of the collagen α1(I) chain induced a sustained migratory response. Other ECM components, such as fibronectin or laminin 421, induced migratory responses that progressively increased over time. A recent study showed that collagen III-deficient fibroblasts produce tumour-permissive collagen matrices that drive cell proliferation and suppress apoptosis in BC cell lines [34].

Therefore, collagen III appears to display context-dependent functions, acting as a tumour-restrictive matrix component in some settings, while supporting migration under specific stromal conditions.

### 3.4. Collagen V

Collagen V is considered a minor component of the fibrillar IM. Nevertheless, its biological role remains poorly understood. Collagen V co-polymerizes with collagen I to regulate fibril assembly and fibril diameter [35]. Therefore, collagen V is likely to play an important role in tumour progression. In fact, a Kyoto Encyclopedia of Genes and Genomes (KEGG) analysis identified that collagen V participates in focal adhesion, regulation of the actin cytoskeleton, and cell-to-ECM interactions [36]. Nevertheless, the prognostic relevance of collagen V in BC remains unclear, as both tumour-promoting and tumour-suppressive functions have been reported, and correlations with clinical outcome remain inconsistent [36,37,38,39].

Collagen V monomers can interact with ECM ligands such as transforming growth factor beta (TGF-β1) and with cell-surface molecules including the proteoglycan (PG) glypican-1, thereby contributing to downstream oncogenic signalling pathways associated with tumour cell adhesion and proliferation. Recent evidence identified *COL5A1* as a driver of tumour growth, metastasis, and doxorubicin resistance in TNBC models [19]. Mechanistically, interleukin 6 (IL-6) secretion by *COL5A1*-overexpressing tumour cells promoted M2 macrophage polarization. In turn, macrophage-derived TGF-β enhanced chemoresistance through activation of the TGF-β/Smad3/COL5A1 axis, thereby establishing a tumour–stroma feedback loop.

These findings suggest that collagen V may function as more than a structural regulator of fibrillogenesis. By linking ECM organization with inflammatory signalling, macrophage polarization and TGF-β-dependent pathways, collagen V emerges as a potential mediator of tumour–stroma crosstalk, particularly in aggressive BC subtypes such as TNBC.

### 3.5. Collagen XI

Compared with other minor fibrillar collagens, collagen XI has emerged as an important component of the tumour ECM in BC. Transcriptomic analyses together with immunohistochemical studies indicate that COL11A1 is predominantly expressed by CAFs, although immunohistochemical evidence also supports expression by subsets of BC cells, suggesting a role in ECM remodelling and tumour–stroma interactions [40,41].

Transcriptomic analyses reported that tumours with prominent lymphocytic infiltration exhibited more than a 100-fold increase in COL11A1 mRNA expression [42]. Transcriptomic and immunohistochemical studies indicate that both CAFs and BC cells are able to produce collagen XI, which may explain the marked variability observed in COL11A1 expression across different stages of disease progression [41,43]. Clinical transcriptomic cohort studies have associated elevated *COL11A1* expression with poor prognosis and altered patterns of tumour immune infiltration, although the underlying biological mechanisms remain under investigation [44].

A recent transcriptomic analysis demonstrated a significant correlation between BC progression and changes in *COL11A1* mRNA expression [45]. Higher *COL11A1* mRNA levels in primary tumours correlate with poor prognosis, whereas lower transcript levels in lymph-node metastases suggest stage-dependent regulation during metastatic dissemination.

In summary, transcriptomic, immunohistochemical, and functional studies suggest that, rather than acting as a passive stromal marker, collagen XI reflects dynamic interactions between CAFs, tumour cells, and immune populations. Its variable expression across disease stages suggests that collagen XI may participate in ECM remodelling programmes that evolve during tumour progression and metastatic dissemination.

### 3.6. Collagens XXIV and XXVII

Compared with other fibrillar collagens, collagens XXIV and XXVII remain poorly characterized in BC. These collagens appear to belong to a distinct evolutionary branch and display structural features associated with ancestral collagen forms [47,48]. In murine models, they form thin fibrillar structures and are predominantly expressed during early embryonic development [49].

Regarding their potential role in cancer, mRNA expression analyses have shown increased *COL24A1* expression in head and neck cancer patients [46]. As for collagen XXVII, its serum concentration was markedly increased in dogs with hemangiosarcoma suffering large metastatic burdens [118]. Although evidence remains limited, further studies are needed to determine whether collagen XXIV and collagen XXVII contribute to early breast tumourigenesis, metastatic dissemination, or adaptation to the metastatic niche.

Overall, fibrillar collagens emerge as the principal determinants of ECM architecture and biomechanical signalling in BC. Although individual collagen types display distinct functions, a common theme is their ability to regulate tissue stiffness, stromal communication, and invasive behaviour through context-dependent interactions with tumour and stromal cells.

## 4. Non-Fibrillar Collagens

Beyond their structural contribution to ECM organization, non-fibrillar collagens actively regulate cell–matrix communication, mechanotransduction, angiogenesis, and immune interactions within the breast TME. Rather than functioning solely as architectural components, these collagens regulate fibril organization, signalling pathways, immune landscapes, and therapy-related phenotypes in a highly dynamic manner.

Among non-fibrillar collagens, FACITs constitute a highly diverse subgroup involved in ECM organization, stability, and cell–matrix communication (Table 2). Instead of forming independent fibrils, FACIT collagens associate with pre-existing collagen networks and regulate tissue architecture, biomechanical signalling, and stromal communication.

Accumulating evidence indicates that several FACIT collagens actively contribute to BC progression. Their reported functions include tumour cell invasion, immune modulation, metastatic dissemination, and therapeutic response. The major FACIT collagens currently implicated in BC include collagens IX, XII, XIV, XVI, XIX, XX, XXI, and XXII.

### 4.1. FACITs

Current evidence supports the involvement of FACIT collagens in metastatic dissemination, immune modulation, and therapy response in BC. Their biological functions extend beyond ECM organization and include the regulation of tumour–stroma communication, mechanotransduction, and subtype-specific signalling programmes.

#### 4.1.1. Collagen IX

Collagen IX contributes to collagen scaffold organization and maintenance of ECM architecture. In BC, altered collagen IX expression has been associated with trastuzumab resistance in HER2-positive tumours, metabolic rewiring in TNBC/basal-like subtypes, and SRY-box transcription factor 10 (SOX10)-associated transcriptional programmes in luminal tumours [119]. These findings suggest that collagen IX may participate in subtype-specific ECM signalling and therapy-resistance programmes.

#### 4.1.2. Collagen XII

Collagen XII is a FACIT collagen characterized by a short collagenous region and a large non-collagenous domain containing multiple interaction modules. Its splice variants display distinct glycosaminoglycan modifications and ligand-binding properties [51]. Beyond its structural functions, collagen XII regulates ECM organization, cell adhesion, and tumour progression by promoting a pro-invasive stromal microenvironment in BC [5]. Recent transcriptomic studies have shown that miRNA-mediated regulation of COL12A1 mRNA expression influences patient prognosis, while chemotherapy agents, including doxorubicin, cisplatin, and tamoxifen, can also modulate COL12A1 transcript level [11,53,120].

Emerging evidence also links collagen XII to tumour-associated immunosuppression. Transcriptomic analyses demonstrated that activation of the kynurenine pathway increases *COL12A1* mRNA expression while promoting regulatory T-cell differentiation and immunosuppressive signalling [121].

Clinical transcriptomic cohort studies have shown that elevated *COL12A1* mRNA expression correlates with M2 macrophage infiltration and TGF-β-associated markers. Moreover, elevated *COL12A1* levels predict poor response to anti-PD-1/PD-L1 immunotherapy [52]. These findings support the potential value of collagen XII as a biomarker of immune remodelling and therapeutic response in BC.

Altogether, these observations position collagen XII as a stromal organizer and immune-modulatory collagen with particular relevance for metastatic progression and immunotherapy resistance.

#### 4.1.3. Collagen XIV

Transcriptomic studies indicate that *COL14A1* displays stage-dependent mRNA expression during BC progression. Although its transcript levels have been reported to decrease from grade I to grade III tumours [54], other transcriptomic studies have reported markedly increased *COL14A1* mRNA expression in BC patients with high lymphatic infiltration, metastasis and, particularly, brain metastasis [55,122]. In these patients, altered expression of collagens III and VI was also observed.

Beyond altered expression, genomic alterations involving *COL14A1* may also contribute to BC biology. The identification of the *COL14A1*-*SKAP1* fusion gene suggests a potential connection between collagen remodelling and immune-cell regulation [123]. This association is particularly relevant considering the role of *SKAP1* in T-cell activation and differentiation. Nevertheless, the prognostic significance of these alterations remains unclear. Interestingly, *EPS15* overexpression has also been associated with favourable prognosis in BC. Moreover, *EPS15* alterations frequently co-occur with *SKAP1* abnormalities [124]. However, the mechanistic relationship between *EPS15*, *SKAP1*, and *COL14A1*-dependent pathways remains unresolved.

#### 4.1.4. Collagen XVI

The functions of collagen XVI in BC remain poorly understood. Similar to other FACIT collagens, collagen XVI contributes to fibrillar ECM organization and mediates cell–ECM interactions through integrin-β1 binding [56,57]. Therefore, altered collagen XVI-dependent adhesion mechanisms may influence tumour invasiveness.

In glioblastoma models, collagen XVI overexpression enhanced cell adhesion without significantly increasing migration [57], whereas in oral cancer, it promoted proliferation through Kindlin-1/integrin-β1 signalling [58].

More recently, collagen XVI was included in an eight-protein signature associated with progression risk in ovarian cancer subtype C1, a molecular subtype defined by its metabolic profile [125]. These effects may partly result from concomitant MMP-9 overexpression, which facilitates ECM degradation and tumour cell migration [59]. In BC, increased mammographic density correlates with elevated collagen XVI expression [68]. However, its precise contribution to ECM organization, tumour stiffness, and disease progression remains insufficiently explored [126].

#### 4.1.5. Collagens XX, XXI, and XXII

The biological functions of collagens XX, XXI, and XXII remain poorly understood. collagen XX is predicted to interact with other collagen fibrils, collagen XXI participates in blood vessel assembly, and collagen XXII contributes to myotendinous junction organization through interactions with integrins α2β1 and α11β1 [67,69,127]. Emerging evidence suggests that these collagens may contribute to several cancer types.

In brain tumour-initiating cells, *COL20A1* was upregulated [128]. *COL21A1* mRNA levels were altered in response to chemotherapy or radiotherapy in ovarian cancer, multiple myeloma, and colorectal cancer [129,130,131]. Circulating collagen XX levels were elevated in sera from patients with several solid cancers and were associated with poor prognosis in pancreatic ductal carcinoma [68].

Interestingly, increased *COL22A1* mRNA levels correlated with lymph node metastases in head and neck cancer patients [47]. In parallel, mutations in *COL22A1* were more frequently detected in pre-treatment samples from TNBC/basal-like BC patients who achieved pathological complete response (pCR) after chemotherapy [21,46]. Therefore, although these collagens display relatively restricted expression patterns, they may still contribute to tumour progression and treatment response in specific cancer contexts.

#### 4.1.6. Collagen XIX

Collagen XIX is a BM-associated FACIT collagen that is highly expressed during embryonic development and subsequently becomes restricted to selected tissues, particularly the nervous system, where it contributes to synaptic maintenance [60]. Consistent with this tissue distribution, altered collagen XIX expression has been reported in neurological diseases. Collagen XIX dysregulation has also been described in several cancer types, including BC, where it is produced by myoepithelial cells [61]. In contrast to several other collagens discussed above, *COL19A1* mRNA expression decreases from the early stages of BC progression. This reduction has been attributed to its susceptibility to proteolytic degradation during BM remodelling [61].

Proteolytic cleavage between the triple-helical collagenous domain 1 (COL1) and the short C-terminal non-collagenous domain 1 (NC1) releases bioactive fragments known as matrikines [62]. Unlike many collagen-derived fragments, these matrikines are generated by plasmin rather than metalloproteinases, and display strong anti-angiogenic activity through vascular endothelial growth factor (VEGF) inhibition [63]. Similarly, NC1-derived fragments reduce phosphorylation and activation of the FAK/PI3K/AKT/mTOR pathway, thereby decreasing tumour cell proliferation and migration [64]. Current evidence suggests that collagen XIX-derived fragments may exert tumour-restrictive effects, whereas reduced *COL19A1* transcript expression may disrupt this protective balance during BC progression.

Further studies are required to clarify the mechanisms underlying collagen XIX degradation in BC and the clinical relevance of plasmin-mediated matrikine release. Preliminary evidence from a multicancer study primarily conducted in patients with non-small cell lung cancer suggests that circulating collagen XIX-derived fragments may have biomarker potential. However, because BC was not specifically investigated, these findings cannot be directly extrapolated to BC, and require dedicated validation in BC cohorts before any clinical relevance can be inferred [65]. Interpreting the available evidence on collagen XIX remains challenging. Its biological effects appear to depend on ECM organization, proteolytic processing, and tumour-stage-specific expression patterns. In BC, reduced collagen XIX expression has been reported during early tumour progression and before BM invasion [61,65,132].

FACIT collagens display remarkable functional diversity in BC, contributing to ECM remodelling, metastatic dissemination, and therapy-associated stromal reprogramming. Their context-dependent functions reinforce the need to interpret FACIT alterations within specific tumour subtypes, stromal niches, and disease stages.

A recurring theme across FACIT collagens is their ability to couple ECM organization with regulatory functions that extend beyond structural support. By modulating fibrillar collagen networks and influencing cell–matrix communication, FACIT members contribute to biomechanical signalling, immune-cell behaviour, and therapy-associated stromal reprogramming. These shared properties position FACIT collagens as emerging indicators of microenvironmental dynamics and potential biomarkers of disease progression and treatment response in BC.

### 4.2. Multiplexins

Because collagen XIX also shares functional similarities with multiplexins through the generation of bioactive anti-angiogenic fragments, it provides a conceptual bridge toward other multiplexin collagens such as collagen XV and collagen XVIII. The growing interest in collagen XIX in BC also supports consideration of collagens XV and XVIII because of their structural and functional similarities. Both collagens have been implicated in ECM homeostasis, angiogenesis, and tumour progression, although through distinct biological mechanisms [77].

Proteolytic processing of collagens XV and XVIII can release their respective C-terminal non-collagenous fragments, namely, the NC10 domain of collagen XV (historically referred to as restin) and endostatin derived from collagen XVIII. Throughout this review, the term restin refers exclusively to the collagen XV-derived NC10 fragment and should not be confused with the unrelated intracellular MAGE-family protein MAGEH1, which has also been designated “restin” in the earlier literature. Collagen XV, for which four isoforms have been described, has been described as a potential anti-angiogenic factor, whereas endostatin also inhibits cell proliferation by binding to heparan sulfate PGs involved in growth factor signalling. The anti-angiogenic activity of the collagen XV-derived NC10 fragment (restin) was initially proposed based primarily on its sequence homology with endostatin rather than on direct functional evidence. Consequently, the biological functions of this extracellular fragment remain incompletely characterized, and should not be extrapolated from studies on the unrelated intracellular protein MAGEH1, historically also termed “restin”. Therefore, the present review discusses only evidence directly related to collagen XV and its NC10-derived fragment [133].

#### 4.2.1. Collagen XV

One study compared full-length collagen XV with several truncated variants lacking either the C-terminal NC10 domain (collagen XV-derived restin) or specific collagenous domains [97]. The results indicated that the effects on tumour growth and cell adhesion were mainly associated with the collagenous region rather than with the NC10/restin domain itself.

In a mammary carcinoma model, *COL15A1* deletion altered the tumour ECM and was associated with increased tumour growth, despite reduced tumour vascularization [98]. These findings indicate that collagen XV may exert context-dependent effects on tumour progression, and that changes in angiogenesis do not necessarily translate into reduced tumour growth. Recombinant human collagen XV has also been shown to regulate cell adhesion and migration, including interactions with collagen I, whereas no comparable adhesion was observed with collagen IV [99].

Overall, the biological functions of collagen XV and its proteolytically released NC10 fragment (restin) remain incompletely characterized. Importantly, this extracellular collagen XV-derived fragment is distinct from the intracellular MAGE-family protein MAGEH1, which has historically also been referred to as “restin”. Although sequence homology with endostatin initially suggested possible anti-angiogenic activity, direct evidence supporting a clinically relevant anti-tumour function of collagen XV-derived restin in BC remains limited. Further studies are required to distinguish the functions of full-length collagen XV from those of its proteolytically released NC10 fragment and to determine their respective contributions to basement-membrane organization, angiogenesis, and tumour progression.

#### 4.2.2. Collagen XVIII

The functional relationship between collagen XVIII and endostatin appears more complex. Collagen XVIII is part of the epithelial and vascular BM, although different isoforms exist depending on the tissue, including a long isoform highly expressed in the liver [100]. This may partly explain why much of the available evidence derives from liver studies. Toxicological studies identified a link between collagen XVIII and integrin-β1/AKT signalling, as deficiency in some of these components increases hepatocyte susceptibility to toxicants [101]. Consequently, collagen XVIII has been proposed to participate in provisional ECM assembly during tissue injury and wound healing.

Recent transcriptomic studies indicate increased *COL18A1* mRNA expression in HER2-positive and TNBC/basal-like BCs [15]. Together, these observations suggest that collagen XVIII contributes to ECM remodelling and proliferative signalling during BC progression.

By contrast, the biological effects of endostatin are considerably better characterized. Endostatin binds strongly to mammary duct BM cells and exerts potent anti-angiogenic activity, which has been exploited in several therapeutic strategies with promising results, particularly in advanced BC and TNBC/basal-like tumours [102,103,104,105,106,107]. These studies support an important role for endostatin in restricting neovascularization, a process particularly relevant in tumours exposed to radiotherapy and radiation necrosis. In addition, vasculogenic mimicry in TNBC/basal-like BC, characterized by the formation of functional vascular-like channels lined by tumour cells, should also be considered when evaluating endostatin-based therapeutic approaches.

An intriguing feature of collagen XVIII is its dual biological behaviour. Whereas full-length collagen XVIII promotes oncogenic signalling through HER2-, EGFR-, and integrin-dependent pathways, its cleavage product endostatin exerts anti-angiogenic and tumour-restrictive effects. This functional dichotomy illustrates how collagen processing can generate biologically distinct molecules with opposing effects on BC progression.

### 4.3. BM Collagens

BM collagens are essential regulators of epithelial integrity, tissue compartmentalization, and tumour–stroma interactions in BC. Beyond their structural functions, BM collagens actively participate in mechanotransduction, EMT, angiogenesis, and metastatic dissemination through dynamic remodelling of the BM architecture. Accordingly, alterations in BM collagen expression and proteolytic processing represent critical events during tumour invasion and metastatic progression.

#### 4.3.1. Collagen IV

Collagen IV is the principal structural component of the BM, particularly within the lamina densa. By forming a mesh-like network that separates the epithelial compartment from the breast stroma, collagen IV maintains tissue compartmentalization. Its degradation is therefore closely associated with tumour invasion and disease progression. This association is supported by previous studies showing that elevated serum levels of C4M (MMP-generated collagen IV fragment), as well as C1M and C3M (MMP-generated collagen I and collagen III fragments, respectively), were correlated with worse prognosis in BC patients [134]. Similar studies reached equivalent conclusions, indicating that circulating collagen IV fragments reflect increased ECM turnover and enhanced metastatic dissemination [70,71].

Mechanistically, collagen IV contributes to tumour progression through signalling pathways involved in EMT, cytoskeletal remodelling, and invasive behaviour [71,99]. Functional experimental studies demonstrated that tumour-associated adipocytes enhance collagen IV production and promote BM remodelling within the TME [81]. Collagen IV overexpression induces E-cadherin downregulation together with upregulation of SNAIL1/2, Stat5, and Sip1 transcription factors, all of which are implicated in tumourigenesis and EMT [72,73]. In addition, DDR1 and DDR2 are activated by collagen IV, which in turn induces further production of MMP-2, and -9, and CD9 through a Src-dependent pathway [74,75]. Src activation is strongly linked to signalling pathways regulating cell proliferation, cytoskeletal reorganization, and tumour cell migration.

These signalling pathways are tightly regulated. In ER+ BC cell lines, adhesion to collagen IV reduces cell migration through the transcription factor AF-1, whereas inhibition of Src activity reverses the suppression of cell invasiveness [76]. These observations may partly explain the comparatively lower invasiveness of ER+ luminal subtypes. Subsequent studies further demonstrated that integrin-αv expression plays a major role in regulating motility in ER + BC cells [77].

Interestingly, binding of collagen IV to integrin-αvβ3 promotes invadopodia formation through dishevelled-associated activator of morphogenesis 1 (DAAM1)-mediated actin polymerization [111]. Invadopodia have attracted considerable attention in recent years because of their central role in tumour invasion and their potential therapeutic tractability [135]. Moreover, collagen IV contributes to vasculogenic mimicry by forming part of the vascular-like channels generated by tumour cells [136].

These observations highlight collagen IV as a central regulator of BM dynamics during BC progression. Beyond its structural role, collagen IV integrates EMT-associated signalling, invadopodia formation, protease activation, and tumour cell plasticity, positioning BM remodelling as a critical step in the transition from local growth to invasive disease.

#### 4.3.2. Collagen XXVIII

Very limited information is currently available regarding the BM collagen XXVIII. It can be abnormally expressed in some types of cancer such as lung cancer or glioblastoma, and it may participate in fibroproliferative disorders [137,138]. Intriguingly, although observing changes in collagen stiffness is a common risk event in BC, there is currently no evidence regarding the expression or functional relevance of collagen XXVIII in BC.

### 4.4. Beaded Filament-Forming Collagens: Collagen VI

Collagen VI is the only beaded collagen identified to date, and forms a disorganized microfibrillar network between epithelial cells in the BM and collagens of the IM. Collagen VI has been implicated in vasculogenic mimicry in melanoma cells [139]. In a pan-cancer proteomic study that included BC cases, two molecular subgroups were proposed based on collagen VI expression and its interacting proteins [140]. Another proteomic-based analysis found that collagen VI dysregulation was identified as a common event in BC, and these alterations were associated with defective organization of cell junctions and EMT [141]. Consequently, collagen VI directly participates in regulatory and organizational ECM remodelling processes. Transcriptomic analyses identified aberrant *COL6A6* transcript expression associated with early pathological stage in BC [142].

Of note, collagen VI further illustrates the importance of stromal compartments in shaping tumour behaviour, as it can be produced by fibroblasts and adipocytes [78,79]. This close interaction between stromal cell populations resembles the intercellular relationships discussed in previous sections, and several ligands act as common mediators. Among these mediators, CCL5 is particularly relevant because it promotes macrophage recruitment while stimulating collagen VI production by fibroblasts and adipocytes [79].

The tumour-promoting consequences of collagen VI upregulation may partly result from the formation of supramolecular linear structures that facilitate tumour cell migration. In addition, the α3(VI) chain of collagen VI acts as a substrate for MMP11, a metalloproteinase secreted by adipocytes, which is required for the correct extracellular folding of collagen VI [78]. The authors showed that adipose-tissue-derived MMP11 promotes tumour progression through localized collagenolytic activity targeting collagen VI at the tumour–adipocyte interface [80].

The biological relevance of collagen VI extends beyond ECM architecture. Its production by fibroblasts and adipocytes places this collagen at the centre of tumour–stroma communication, linking adipose tissue remodelling, inflammatory signalling, and migratory niches that may facilitate tumour progression.

### 4.5. Anchoring Fibril-Forming Collagens: Collagen VII

Similar to collagen VI, collagen VII is the only representative of its collagen subgroup. In this context, collagen VII functions as an ECM-to-ECM anchoring fibre between the lamina densa layer and the major collagens of the IM, collagens I and III. Based on currently available evidence, in which it has been found to be produced preferentially by keratinocytes and fibroblasts, it may represent a reinforcing collagen predominantly found in partially keratinized epithelia. This would explain its absence in several types of cancer, except for bladder cancer, squamous carcinomas of the lung, cervical cancers, and head and neck cancer [82].

Very limited evidence regarding collagen VII dysregulation in BC is currently available, although sporadic expression has been reported. Moreover, most available evidence is indirect and does not derive from BC-specific functional studies focused on collagen VII. Nevertheless, the available evidence suggests that collagen VII expression would have a tumour-suppressive role, as inactivating-hypermethylation and *COL7A1* gene mutations were found in BC, and both events correlated with poor prognosis of those patients [84,85].

A recent study hypothesizes that collagen VII is a favourable prognostic marker in BC, as it was associated with reduced proliferation of the BC cell line MCF-7 in vitro, as well as with several positive prognostic markers in human BC tissue [83].

### 4.6. Network-Forming Collagens

Although collagens VIII and X both belong to the network-forming collagen subgroup, their biological functions are fundamentally different. Collagen VIII forms hexagonal lattice-like networks and contributes to vascular architecture and angiogenic regulation, whereas collagen X assembles into short-chain networks and participates in physiological cartilage mineralization. Therefore, their biological functions are fundamentally distinct.

#### 4.6.1. Collagen VIII

Few studies have investigated the role of collagen VIII in BC, although available evidence supports its involvement in angiogenesis and vascular remodelling [86]. Transcriptomic analyses demonstrated that BC patients had higher *COL8A1* mRNA expression levels than healthy volunteers. Although *COL8A1* showed only moderate discriminatory capacity between these cohorts, elevated mRNA levels identified a subgroup of patients with poorer prognosis [87].

Although further research is required, collagen VIII overexpression may promote vascular remodelling within tumour-associated vessels, and may facilitate metastatic dissemination, a function comparable with other collagens. A possible association between *COL8A2* and Procollagen C-proteinase enhancer protein (PCOLCE), an enhancer of collagen maturation, has been reported in relation to bone metastatic disease. However, the available evidence derives mainly from osteosarcoma, and its relevance to BC remains to be established. In that study, increased PCOLCE mRNA expression was associated with poorer prognosis and a higher risk of bone metastasis, whereas *COL8A2* and *COL10A1* were also found to be overexpressed [143].

#### 4.6.2. Collagen X

Compared with collagen VIII, more information is available regarding the role of collagen X in BC. Immunohistochemical studies have demonstrated increased collagen X protein expression within the vascular network of several cancer types, including BC [88,89]. Furthermore, its expression co-localizes with that of elastin fibres, another major component of the IM and blood vessel walls [90].

Notably, the prognostic relevance of collagen X appears to be strongly subtype-dependent, such that it is selectively expressed in ER+ and HER2+ subtypes, whereas the correlation with the TNBC/basal-like subtype is less well defined [90,91]. Patients with these subtypes and with collagen X overexpression had a worse prognosis. Collagen X may also serve as a biomarker for predicting response to neoadjuvant anti-HER2 therapy in HER2-positive and ER-positive BC subtypes, and its predictive value appears to increase when tumour-infiltrating lymphocytes (TILs) are also considered, as patients with collagen X overexpression and low lymphocyte infiltration had worse response to this therapy [13,17].

In this sense, the importance of the TME and the composition of the matrisome is clearly evident. A recent study showed that knocking down COL10A1 inhibited the PI3K/AKT signalling pathway via its direct interacting protein integrin-β1 (ITGB1), thereby restraining TNBC cell proliferation, migration, invasion, and suppressing tumour growth and lung metastasis [20].

### 4.7. MACITs

MACIT is a non-fibrillar collagen subgroup that includes four collagens (collagens XIII, XVII, XXIII, and XXV) with a common structure: a short cytosolic N-terminal domain, the transmembrane domain, and a larger C-terminal ectodomain that contains the collagenous domains. Their transmembrane localization enables MACIT collagens to function as molecular interfaces between intracellular signalling pathways and the ECM.

Among them, collagens XIII and XVII are the best-characterized MACIT collagens in BC, although alterations in other MACIT members were detected in other cancers, or even different diseases. Collagen XIII induces integrin-β1 expression, enhances cancer cell stemness, and, notably, promotes anoikis resistance, thereby enabling tumour cell survival after detachment from the primary tumour site, a prerequisite for metastatic dissemination [92]. Inhibition of either integrin-β1 or collagen XIII reduced metastatic potential.

On the other hand, collagen XVII downregulation in advanced stages of BC is common, and it correlates with worse prognosis [93]. Likewise, axillary lymph node metastasis from TNBC/basal-like tumours had a comparatively lower *COL17A1* expression [94]. Together, these observations support the existence of a regulatory mechanism for collagen XVII in a p53-dependent manner, as the expression of this collagen is involved in the epidermal development [95].

An alternative mechanism for this tumour-related suppressing activity of collagen XVII in BC was proposed after finding its expression down-regulates the activity of several effectors of the AKT/mTOR signalling pathway [96]. This downregulation appears to be mediated by the hypermethylation of the *COL17A1* promoter [93].

Overall, non-fibrillar collagens emerge as central coordinators of ECM remodelling and tumour–stroma crosstalk in BC. Beyond their structural functions, these collagens actively influence mechanotransduction, immune modulation, angiogenesis, metastatic dissemination, and therapeutic resistance through highly dynamic tumour–stroma interactions. Their remarkable functional diversity reinforces the concept that collagen remodelling actively drives BC progression rather than merely representing a passive consequence of tumour growth.

## 5. Unclassified Collagens: Collagen XXVI

Finally, collagen XXVI cannot currently be assigned to any established collagen subgroup because of its unique sequence characteristics and supramolecular organization. Its expression has mainly been detected in the ovary and testis, as well as in neonatal genital tissues, where it is present at higher levels. Accordingly, it has been proposed to participate in embryonic development and tissue modelling during growth [108].

Very limited information is currently available regarding collagen XXVI, and no studies have specifically investigated its role in BC. A transient supportive role during IM development has also been observed in perinatal kidney extracts in a recent study [144]. In addition, collagen XXVI appears to be tightly regulated in lymphocytes, where it may participate in cell–cell adhesion and intercellular communication [145].

These observations are particularly interesting because proteins involved in embryogenesis are often re-expressed during tumourigenesis and tumour progression. Whether collagen XXVI undergoes similar re-expression during BC progression remains completely unexplored.

## 6. Collagen-like Domain Proteins

Although the primary focus of this review is the human collagen family, several proteins containing collagen-like domains have also been implicated in BC. Unlike structural collagens, these molecules do not constitute major ECM components, but rather regulate immune responses, ECM homeostasis, and tumour–stroma communication, thereby indirectly influencing collagen-dependent processes within the TME. Because their contribution is complementary to that of canonical collagens, only the principal proteins currently associated with BC are summarized below.

Complement component C1q has attracted considerable interest because of its dual role in innate immunity and tumour biology [146]. Although C1q may exert either tumour-promoting or tumour-suppressive effects depending on the cancer type, most evidence in BC supports a protective role through regulation of immune surveillance, angiogenesis, and complement activation [147,148]. C1q also interacts with ADAM28, a metalloendopeptidase implicated in BC progression and metastasis [149]. Formation of the ADAM28–C1q complex reduces the pro-apoptotic activity of C1q, suggesting that the biological effects of both proteins depend on their spatial distribution within the TME [150,151]. Beyond its immunological functions, C1q also contributes to angiogenic regulation [152] and may participate in the therapeutic efficacy of trastuzumab/pertuzumab-based anti-HER2 therapy by enhancing complement-mediated immune responses [16].

Other collagen-like lectins involved in innate immunity have also been associated with BC. Ficolins (ficolin-1, -2, and -3) participate in complement activation through the lectin pathway and regulate macrophage polarization and antitumour immune responses [146]. Reduced circulating ficolin levels have been reported in BC patients, and experimental evidence suggests that restoration of ficolin activity may enhance macrophage- and lymphocyte-mediated antitumour responses [153,154]. Likewise, altered ficolin levels have also been associated with infection susceptibility in haematological malignancies, highlighting their broader immunological relevance [155]. Although this aspect remains poorly investigated in BC, infectious complications are clinically relevant in these patients [156].

Similarly, mannose-binding lectin (MBL2) has been linked to BC susceptibility and treatment outcome [157,158,159]. Genetic polymorphisms affecting MBL2 expression have been associated with differences in cancer susceptibility and increased risk of severe chemotherapy-related infections [160]. In addition, elevated circulating MBL2 levels have been associated with poorer survival and failure to achieve pCR following neoadjuvant treatment, although these findings probably reflect systemic tissue damage rather than a direct tumour-promoting role [161].

Ectodysplasin, a tumour necrosis factor-like ligand involved in mammary gland development, regulates fibroblast growth factor 20 (FGF20) and NF-κB signalling pathways that control epithelial morphogenesis [162,163,164].

Consistent with its developmental functions, ectodysplasin signalling has also been implicated in BC. A recent study demonstrated increased activation of the ectodysplasin receptor in ER-negative BC subtypes, which was associated with increased tumour burden and more extensive squamous metaplasia [165]. Recent studies have also linked increased ectodysplasin receptor activation with ER-negative BC, suggesting that developmental pathways may become reactivated during tumour progression [166].

Finally, elastin microfibril interface proteins (emilins) contribute to ECM organization by connecting cells with elastic fibres and regulating transforming growth factor beta (TGF-β) bioavailability [167,168]. Reduced EMILIN-1 expression has been associated with increased tumour growth and metastatic dissemination, supporting the concept that alterations in elastin-associated matrix components cooperate with collagen remodelling during BC progression.

The evidence discussed throughout this review highlights that collagens and collagen-associated pathways exert clinically relevant and subtype-dependent effects in BC progression, immune modulation, metastatic dissemination, and therapeutic response. To facilitate comparison across collagen families, Table 3 summarizes the current clinical and translational evidence supporting their involvement in BC. Prognostic associations, predictive biomarker evidence, and experimental mechanisms are presented separately, together with the principal source and overall strength of the available evidence. This organization allows a clearer distinction between clinically supported observations and mechanistic findings derived from experimental studies.

## 7. Conclusions and Future Perspectives

Collagens have emerged as central regulators of BC (BC) biology, extending far beyond their classical structural role within the ECM. The evidence summarized throughout this review demonstrates that collagen remodelling is a dynamic and spatially organized process that influences virtually every stage of disease progression, including tumour initiation, stromal activation, local invasion, metastatic dissemination, immune modulation, and therapeutic response. Through coordinated alterations in collagen deposition, fibrillar organization, enzymatic crosslinking, and proteolytic remodelling, the collagenome actively regulates ECM stiffness, mechanotransduction, and tumour–stroma communication.

Importantly, the biological functions of collagens are highly context-dependent. Individual collagen types may exert tumour-promoting or tumour-restrictive effects depending on their supramolecular organization, anatomical localization, cellular source, tumour subtype, and disease stage. Fibrillar collagens primarily regulate IM remodelling and tissue mechanics, whereas BM collagens preserve epithelial compartmentalization and influence invasion. FACIT, MACIT, and multiplexin collagens further increase this complexity by regulating collagen fibrillogenesis, cell–matrix signalling, immune-cell interactions, and the generation of biologically active matrikines. These observations emphasize that the collagenome should be regarded as an integrated and highly dynamic signalling network rather than as a collection of isolated structural proteins.

From a translational perspective, collagen-associated alterations provide multiple opportunities for improving BC management. Tissue collagen signatures, circulating collagen-derived fragments, and imaging-based assessment of collagen architecture may contribute to more accurate prognostic stratification and prediction of therapeutic response. Likewise, therapeutic strategies targeting collagen deposition, LOX-mediated crosslinking, matrix stiffness, collagen–integrin signalling, CAF activation, or ECM remodelling have emerged as promising approaches, particularly for aggressive BC subtypes such as HER2-positive and triple-negative BC. The current translational landscape of collagen-derived biomarkers and collagen-targeted therapeutic strategies discussed throughout this review is summarized in Table 4. Nevertheless, the context-dependent nature of collagen biology indicates that future therapeutic strategies should be guided by tumour subtype, spatial ECM organization, and molecular context.

Despite these advances, several important challenges remain. Many collagen types remain poorly characterized in BC, particularly collagens XX, XXI, XXII, XXIII, XXV, XXVI, and XXVIII, while the temporal evolution of collagen remodelling throughout tumour initiation, progression, metastasis, and treatment response is still incompletely understood. Furthermore, the molecular mechanisms linking collagen architecture with mechanotransduction, immune regulation, metabolic adaptation, and therapeutic resistance require further clarification.

Future research should therefore move beyond the analysis of individual collagen molecules and adopt systems-level approaches integrating spatial transcriptomics, single-cell sequencing, quantitative proteomics, advanced imaging, biomechanical analyses, and clinically annotated patient cohorts. Such multidisciplinary strategies will improve our understanding of collagen-producing cell populations and their interactions with cancer cells, CAFs, adipocytes, endothelial cells, and immune infiltrates within the TME. In parallel, prospective multicentre studies will be required to validate collagen-derived biomarkers and determine the clinical utility of collagen-targeted therapies.

Overall, the collagenome represents one of the most biologically informative and therapeutically actionable components of the breast TME. A deeper understanding of collagen diversity and ECM remodelling will not only improve our knowledge of BC biology, but also facilitate the development of more precise biomarkers and innovative stromal-targeted therapeutic strategies, ultimately contributing to more personalized management of patients with BC.

As summarized in Table 4, although numerous collagen-targeted strategies have demonstrated encouraging activity in preclinical BC models, most remain at an early stage of development. Many approaches have only been evaluated in cell culture systems or animal models, whereas robust prospective clinical validation is still limited. Moreover, previous attempts to therapeutically target the tumour stroma, particularly broad-spectrum MMP inhibitors, have shown limited efficacy and unacceptable toxicity in clinical trials, highlighting the complexity of ECM biology. Likewise, inhibition of collagen crosslinking, integrin signalling, FAK, DDRs, or CAFs may interfere with physiological ECM remodelling, wound healing, and tissue homeostasis. Consequently, these strategies should currently be regarded as promising investigational approaches rather than established therapeutic options. Future studies will need to identify appropriate patient populations, optimize treatment combinations, and demonstrate clinical benefit in prospective trials before routine clinical implementation.

## 8. Literature Search Strategy

This narrative review was based on literature retrieved from PubMed, Scopus, and Web of Science. The search was primarily focused on publications available up to June 2026, and used combinations of keywords including *breast cancer*, *extracellular matrix*, *collagen*, *collagenome*, *tumour microenvironment*, *mechanotransduction*, *cancer-associated fibroblasts*, *metastasis*, *angiogenesis*, *immune modulation*, *biomarkers*, and *therapy*. Additional relevant publications were identified through manual screening of the reference lists of selected articles. Priority was given to original research articles, systematic reviews, meta-analyses, and high-quality review papers published in peer-reviewed journals, with particular emphasis on recent studies while also including seminal publications considered essential for understanding collagen biology and its role in BC progression. The final selection of references was based on their scientific relevance and their contribution to the biological, mechanistic, translational, and clinical understanding of collagen families in BC. Studies not directly related to BC, collagen biology, or the TME, as well as publications lacking sufficient mechanistic or translational relevance, were not considered.

## Figures and Tables

**Table 1:** Collagen-associated ECM features reported across clinicopathological BC subgroups. The included studies differ in design, methodology, cohort composition, and sample size; therefore, the summarized associations should be interpreted as evidence-based trends rather than universally established characteristics of each subgroup.

Clinicopathological BC Subgroup	Main Collagen-Associated Features	Evidence Type	Evidence Level	References
HR+/HER2−	Altered expression and organization of fibrillar and BM collagens have been reported in selected HR-positive cohorts and experimental models. These alterations are associated with stromal activation, ECM remodelling, tumour cell migration, and possible differences in prognosis or treatment response	Human tumour cohorts supported by experimental ER-positive BC models	Moderate	[11,12,13]
HER2+	Selected collagens and collagen-dependent signalling pathways have been associated with resistance to HER2-targeted therapy, tumour–ECM signalling, and treatment response	Retrospective clinical cohorts together with experimental HER2-positive BC models	Moderate	[14,15,16,17]
TNBC	Extensive collagen remodelling, LOX-dependent matrix stiffening, and altered expression of several collagens have been associated with EMT, immune modulation, metastatic dissemination, and resistance to chemotherapy or immunotherapy	Human tumour cohorts, transcriptomic studies, and experimental TNBC models	Moderate–High	[18,19,20,21]

**Evidence level:** High, consistent findings from multiple independent human BC studies supported by experimental evidence; moderate, limited or heterogeneous human evidence supported by experimental BC models; the strength of evidence differs among the individual collagen types and mechanisms summarized for TNBC. References listed correspond to the principal studies supporting the associations summarized in each row; the additional supporting literature is discussed throughout the main text.

**Table 2:** Classification of human collagen types and their reported implication in BC. Human collagen types are classified according to their supramolecular organization and biological functions in the breast ECM. The table summarizes collagen subgroups, gene symbols, and experimental evidence regarding their implication in BC progression.

Collagen Name	Gene Symbol	Trimer Composition	Evidence Level	References
**Fibril-forming collagens**				
Collagen I	*COL1A1*, *COL1A2*	[α1(I)]_2_α2(I)/[α1(I)]_3_	High	[12,24,25,26,27,28,29]
Collagen II	*COL2A1*	[α1(II)]_3_	Moderate	[22,23,30,31]
Collagen III	*COL3A1*	[α1(III)]_3_	High	[28,29,32,33,34]
Collagen V	*COL5A1*, *COL5A2*, *COL5A3*	[α1(V)]_2_α2(V)/[α1(V)]_3_/ [α1(XI)α1(V)α3(XI)]	High	[19,35,36,37,38,39]
Collagen XI	*COL11A1*, *COL11A2*, *COL2A1*	[α1(XI)α2(XI)α3(XI)]/ [α1(XI)α1(V)α3(XI)] *	High	[13,17,40,41,42,43,44,45]
Collagen XXIV	*COL24A1*	[α1(XXIV)]_3_	Indirect	[46]
Collagen XXVII	*COL27A1*	[α1(XXVII)]_3_	Indirect	[47,48,49]
**Non-fibril-forming collagens**				
**FACITs collagens**				
Collagen IX	*COL9A1*, *COL9A2*, *COL9A3*	α1(IX)α2(IX)α3(IX)	Moderate	[22,23,30,50]
Collagen XII	*COL12A1*	[α1(XII)]_3_	High	[5,10,51,52]
Collagen XIV	*COL14A1*	[α1(XIV)]_3_	Moderate	[53,54,55]
Collagen XVI	*COL16A1*	[α1(XVI)]_3_	Low	[56,57,58,59]
Collagen XIX	*COL19A1*	[α1(XIX)]_3_	Moderate	[60,61,62,63,64,65,66]
Collagen XX	*COL20A1*	[α1(XX)]_3_	Indirect	[67,68]
Collagen XXI	*COL21A1*	[α1(XXI)]_3_	Indirect	[69]
Collagen XXII	*COL22A1*	[α1(XXII)]_3_	Low	[46,69]
**BM collagens**				
Collagen IV	*COL4A1*, *COL4A2*, *COL4A3*, *COL4A4*, *COL4A5*, *COL4A6*	[α1(IV)]_2_α2(IV)/ α3(IV)α4(IV)α5(IV)/ [α5(IV)]_2_α6(IV)	High	[70,71,72,73,74,75,76,77]
Collagen XXVIII	*COL28A1*	[α1(XXVIII)]_3_	Indirect	[69]
**Beaded filament-forming collagens**				
Collagen VI	*COL6A1*, *COL6A2*, *COL6A3*	α1(VI)α2(VI)α3(VI)	High	[78,79,80,81]
**Anchoring fibril-forming collagens**				
Collagen VII	*COL7A1*	[α1(VII)]_3_	Low	[82,83,84,85]
**Network-forming collagens**				
Collagen VIII	*COL8A1*, *COL8A2*	[α1(VIII)]_2_α2(VIII)/ α1(VIII)[α2(VIII)]_2_/ [α1(VIII)]_3_/[α2(VIII)]_3_	Moderate	[86,87]
Collagen X	*COL10A1*	[α1(X)]_3_	High	[20,88,89,90,91]
**MACIT collagens**				
Collagen XIII	*COL13A1*	[α1(XIII)]_3_	High	[92]
Collagen XVII	*COL17A1*	[α1(XVII)]_3_	High	[93,94,95,96]
Collagen XXIII	*COL23A1*	[α1(XXIII)]_3_	Low	-
Collagen XXV	*COL25A1*	[α1(XXV)]_3_	Indirect	-
**Multiplexins**				
Collagen XV	*COL15A1*	[α1(XV)]_3_	Moderate	[61,97,98,99]
Collagen XVIII	*COL18A1*	[α1(XVIII)]_3_	High	[15,100,101,102,103,104,105,106,107]
**Unclassified**				
Collagen XXVI	*COL26A1*	[α1(XXVI)]_3_	Indirect	[108]

Hybrid type V/XI heterotrimers have been reported in selected human tissues because collagen V and collagen XI belong to the same fibrillar collagen subfamily and may share α-chain assembly. * The α3(XI) and α1(II) chains are encoded by the same gene, *COL2A1*, but undergo different post-translational processing. **Evidence level:** high, multiple independent studies in human BC cohorts with supporting experimental evidence; moderate, limited human BC evidence supported by experimental BC models; low, evidence mainly derived from experimental models or isolated BC studies; indirect, evidence derived predominantly from other tumour types, developmental biology, or veterinary studies. Representative references are provided to identify the principal studies supporting each summarized association. The additional supporting literature is discussed in the corresponding sections of the main text.

**Table 3:** Clinical and translational evidence supporting the role of collagen families in BC. Prognostic evidence refers to reported associations with patient outcome, clinicopathological characteristics, or disease progression. Predictive biomarker evidence summarizes studies evaluating treatment response or therapeutic stratification. Experimental mechanism describes biological functions demonstrated in BC cell lines, animal models, or mechanistic studies. The strength of evidence reflects the overall consistency of the available literature.

Collagen/Factor	Subgroup	Prognostic Evidence	Predictive Biomarker Evidence	Experimental Mechanism	Evidence Source	Overall Evidence	References
Collagen I (*COL1A1*/ *COL1A2*)	Fibrillar; IM	Low COL1A1 linked to poor prognosis	May predict chemotherapy sensitivity	CAF-mediated ECM stiffening, mechanotransduction, and invasion	Human cohorts; in vitro	Moderate	[12,24,25,26,27,117]
Collagen II (*COL2A1*)	Fibrillar	No consistent prognostic value	Reduced response to neoadjuvant anti-HER2 therapy	Integrin-β1/Src activation; collagen inhibition restores sensitivity	Clinical studies; in vitro	Moderate	
Collagen III	Fibrillar	Context-dependent prognostic value	No validated evidence	Regulates dormancy, apoptosis, and migration	Human cohorts; in vitro	Moderate	[28,29,32,33,34]
Collagen V	Fibrillar	*COL5A1* associated with metastasis	Associated with doxorubicin resistance in TNBC	IL-6/TGF-β signalling, M2 polarization, and chemoresistance	Human cohorts; in vitro; in vivo	Moderate	[19,35,36,37,38,39]
Collagen XI	Fibrillar	High *COL11A1* associated with poor prognosis	Possible radiotherapy biomarker	CAF-mediated ECM remodelling and immune regulation	Human cohorts; in vitro; in vivo	High	[40,41,42,43,44,45,169]
Collagen IX	FACIT	No consistent prognostic value	Associated with trastuzumab resistance	ECM signalling and metabolic adaptation	Clinical studies; in vitro	Moderate	[119,170,171]
Collagen XII	FACIT	High *COL12A1* associated with poor prognosis	Predicts poor response to anti-PD-1/PD-L1 therapy	Stromal remodelling, immune suppression, and TGF-β signalling	Human cohorts; in vitro; in vivo	High	[11,52,53,120,121,172]
Collagen XIV	FACIT	Associated with lymphatic and brain metastasis	No validated evidence	Dynamic stromal remodelling; immune-related functions	Human cohorts; in vivo	Moderate	[55,122,123,124]
Collagen XVI	FACIT	Associated with high mammographic density	No validated evidence	Integrin-mediated ECM remodelling	Human cohorts; in vitro	Low	[56,57,58,59,125,126]
Collagen XIX	FACIT	Reduced during tumour progression	No validated evidence	Anti-angiogenic matrikines suppress VEGF signalling	Experimental models	Moderate	[60,61,62,63,64,65,132]
Collagen VI	Beaded filament	Associated with poor prognosis and EMT	Potential biomarker	Fibroblast/adipocyte-derived ECM remodelling	Human cohorts; in vitro; in vivo	Moderate	[78,79,80,139,140,141,142]
Collagen VII	Anchoring fibrils	Epigenetic alterations linked to poor prognosis	No validated evidence	Putative tumour suppressor	Human cohorts; in vitro	Low	[82,83,84,85]
Collagen VIII/ PCOLCE	Network-forming	Limited prognostic evidence	Potential biomarker	Angiogenesis and vascular remodelling	Human cohorts; in vitro	Moderate	[86,87,143]
Collagen X	Network-forming	High *COL10A1* associated with poor prognosis	Predicts response to neoadjuvant anti-HER2 therapy	ITGB1/PI3K/AKT signalling and immune modulation	Human cohorts; clinical studies; in vitro	High	[13,17,20,88,89,90,91]
Collagen XIII	MACIT	Associated with metastatic competence	No validated evidence	Anoikis resistance, integrin signalling, and stemness	Human cohorts; in vitro	Moderate	[92]
Collagen XVII	MACIT	Reduced expression associated with advanced disease	No validated evidence	p53-regulated tumour suppressor; inhibits AKT/mTOR	Human cohorts; in vitro	Moderate	[93,94,95,96]
Collagen XVIII	Multiplexin	High *COL18A1* in aggressive HER2-positive and basal-like B	Endostatin evaluated as therapeutic biomarker	Full-length collagen XVIII promotes HER2/EGFR signalling; endostatin is anti-angiogenic	Human cohorts; clinical studies; in vitro; in vivo	High	[15,100,101,102,103,104,105,106,107]

**Evidence level:** High, consistent findings from multiple independent human BC studies supported by complementary experimental evidence; moderate, limited or heterogeneous human BC evidence supported by experimental BC models; low, evidence derived predominantly from isolated clinical studies or preclinical models requiring further independent validation. **Prognostic evidence** refers to associations with patient outcome, survival, or clinicopathological characteristics. **Predictive biomarker evidence** refers to associations with treatment response, patient stratification, or therapeutic decision-making. **Experimental mechanism** summarizes biological mechanisms demonstrated in BC experimental models. **Evidence source:** Human cohorts, observational studies performed in BC patient samples; **clinical studies**, investigations evaluating treatment response or therapeutic biomarkers; **in vitro**, mechanistic studies performed in BC cell lines; **in vivo**, preclinical animal models. The evidence level reflects the overall consistency of the currently available literature rather than the importance of the biological effect. References listed correspond to the principal studies supporting the associations summarized in each row; the additional supporting literature is discussed throughout the main text.

**Table 4:** Current translational status of collagen-targeted therapeutic strategies in BC. The table summarizes the biological rationale, current level of evidence, stage of development, principal limitations, and safety concerns associated with collagen-directed therapeutic approaches. Although several strategies have demonstrated encouraging activity in preclinical BC models, most remain investigational and require prospective clinical validation before routine clinical implementation.

Therapeutic Strategy	Biological Rationale	Current Evidence	Development Stage	Main Limitations/Safety Concerns	Translational Perspective	References
Endostatin (*COL18A1*-derived fragment)	Inhibits angiogenesis and tumour vascularization	Clinical and extensive preclinical evidence; modest, context-dependent benefit	Early-phase clinical trials/exploratory evidence	Variable efficacy; no validated predictive biomarkers; not established in BC	Promising adjunctive strategy requiring patient selection and combination therapies	[15,104,105,106]
LOX inhibition	Reduces collagen crosslinking, ECM stiffness, and mechanotransduction	Strong preclinical evidence; minimal clinical validation	Cell studies + animal studies	Possible effects on connective tissue remodelling and wound healing	Promising experimental approach requiring clinical validation	[18,109]
FAK/Src inhibition	Blocks collagen-mediated mechanotransduction	Strong preclinical evidence; limited early clinical evaluation	Animal studies + early clinical trials	Limited monotherapy efficacy; toxicity under evaluation	Investigational strategy; not yet suitable for routine use	[14,111]
Integrin-targeted therapies	Inhibit tumour-cell adhesion, migration, and ECM signalling	Extensive preclinical evidence; inconsistent clinical results	Cell studies + animal studies + limited clinical trials	Pathway redundancy; possible effects on tissue repair and immunity	Investigational approach requiring biomarker-guided selection	[14,111,115]
DDR1/DDR2 inhibition	Blocks collagen receptor signalling involved in invasion, EMT, and therapy resistance	Predominantly preclinical evidence	Cell studies + animal studies	No prospective validation; long-term safety unknown	Promising preclinical target	[111,114,115]
CAF-directed therapies	Reduce collagen deposition and stromal remodelling	Mainly experimental evidence	Cell studies + animal studies	CAF heterogeneity; possible impairment of tissue repair	Requires selective targeting before translation	[10,11,116]
Broad-spectrum MMP inhibition	Prevents collagen degradation and ECM remodelling	Evaluated in multiple clinical trials with native outcomes	Clinical trials	Poor efficacy, musculoskeletal toxicity, and limited specificity	Broad inhibition abandoned; focus to selective MMPs targeting	[6,78]
Collagen-directed drug delivery	Exploits collagen-rich ECM for targeted drug delivery	Proof-of-concept experimental evidence	Cell studies + animal studies	Manufacturing complexity, tumour heterogeneity, and lack of clinical validation	Innovative platform at an early translational stage	[1,2,113]

**Development stage:** Cell studies, evidence derived exclusively from in vitro experiments; animal studies, validated in preclinical animal models; retrospective human cohorts, observational studies using existing patient cohorts; prospective validation, prospective observational studies; clinical trials, interventional clinical studies. The assigned stage corresponds to the highest level of evidence currently available for each strategy. **Translational perspective:** Strategies described as investigational, experimental, or early translational should not be interpreted as established clinical therapies, but rather as approaches currently undergoing preclinical or clinical evaluation. References listed correspond to the principal studies supporting the associations summarized in each row; the additional supporting literature is discussed throughout the main text.

## Data Availability

No new data were created or analyzed in this study. Data sharing is not applicable to this article.

## Abbreviations

The following abbreviations are used in this manuscript:

ADAM	A disintegrin and metalloproteinase
AKT	Protein kinase B
AP-1	Activator protein-1
BC	Breast cancer
BM	Basement membrane
CAF	Cancer-associated fibroblast
DAAM1	Dishevelled-associated activator of morphogenesis 1
DDR	Discoidin domain receptor
DDR1/DDR2	Discoidin domain receptor 1/2
ECM	Extracellular matrix
EGFR	Epidermal growth factor receptor
EMT	Epithelial–mesenchymal transition
ER	Estrogen receptor
ERK	Extracellular signal-regulated kinase
FACIT	Fibril-associated collagens with interrupted triple helices
FAK	Focal adhesion kinase
FGF20	Fibroblast growth factor 20
HER2	Human epidermal growth factor receptor 2
HIF-1α	Hypoxia-inducible factor-1α
IL-6	Interleukin 6
ILK	Integrin-linked kinase
IM	Interstitial matrix
ITGB1	Integrin beta-1
KEGG	Kyoto Encyclopedia of Genes and Genomes
LOX	Lysyl oxidase
LOXL2	Lysyl oxidase-like 2
MACIT	Membrane-associated collagens with interrupted triple helices
MAPK	Mitogen-activated protein kinase
MBL2	Mannose-binding lectin 2
MMP	Matrix metalloproteinase
MRI	Magnetic resonance imaging
mTOR	Mechanistic target of rapamycin
NC1	Non-collagenous domain 1
NF-κB	Nuclear factor kappa-light-chain-enhancer of activated B cells
pCR	Pathological complete response
PD-1	Programmed cell death protein 1
PD-L1	Programmed death-ligand 1
PG	Proteoglycan
PI3K	Phosphoinositide 3-kinase
SHG	Second harmonic generation
ROCK	Rho-associated coiled-coil containing protein kinase
SOX10	SRY-box transcription factor 10
Src	Proto-oncogene tyrosine-protein kinase Src
TACSs	Tumour-associated collagen signatures
TAZ	Transcriptional co-activator with PDZ-binding motif
TGF-β	Transforming growth factor beta
TILs	Tumour-infiltrating lymphocytes
TME	Tumour microenvironment
TNBC	Triple-negative breast cancer
VEGF	Vascular endothelial growth factor
YAP	Yes-associated protein
